# Occurrence, Properties, Applications and Analytics of Cytosine and Its Derivatives

**DOI:** 10.3390/molecules30173598

**Published:** 2025-09-03

**Authors:** Mariusz Kluska, Joanna Jabłońska, Dorota Prukała, Wiesław Prukała

**Affiliations:** 1 Faculty of Natural Sciences, University of Siedlce, 54 3-Maja Str., 08-110 Siedlce, Poland; 2 Faculty of Chemistry, Adam Mickiewicz University, Uniwersytetu Poznanskiego 8, 61-614 Poznan, Poland; dorota.prukala@amu.edu.pl (D.P.); wieslaw.prukala@amu.edu.pl (W.P.)

**Keywords:** analytics, applications, biological activity, cytosine, cytosine derivatives

## Abstract

Cytosine and its derivatives are an important research topic in the fields of bioorganic chemistry, molecular biology and medicine due to their key role in the structure and function of nucleic acids. The article provides a detailed overview of the natural occurrence of cytosine, its biosynthetic and degradation pathways in living organisms, as well as its physicochemical and chemical properties. Particular attention was paid to the biological activity and therapeutic applications of cytosine derivatives, including their use in cancer, antiviral and epigenetic therapy. The analytical section describes high-performance liquid chromatography techniques as a major tool for identifying and determining cytosine and its derivatives in biological samples. Examples of separation conditions, column selection, mobile phases and detection parameters for these compounds are presented. The article also provides chemical structures, graphs, comparative tables and an up-to-date review of the scientific literature, presenting a comprehensive overview of the topic, including biological, chemical and analytical aspects.

## 1. Introduction

**Figure 1:**
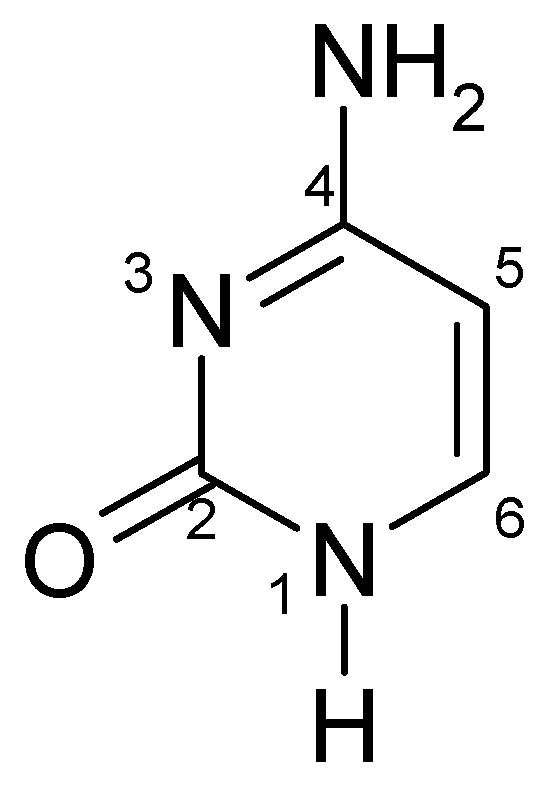
Structural formula of cytosine.

Cytosine (C) is one of the four nitrogenous bases of nucleic acids, alongside adenine, guanine and thymine (in DNA) and uracil (in RNA) [1,2]. It was first isolated from calf thymus tissue in 1894 by Albrecht Kossel and Albert Neumann [3]. Structurally, cytosine is a pyrimidine derivative with a heterocyclic, aromatic ring containing two functional groups: an amine group at position 4 and a carbonyl group (oxo group) at position 2 (Figure 1) [4,5].

In DNA, cytosine occurs as 2′-deoxycytidine (a nucleoside with deoxyribose), and in RNA as cytidine (a nucleoside with ribose). In both cases, it forms complementary pairs with guanine through three hydrogen bonds (in the Watson–Crick model) that stabilise the double helix structure. Cytosine therefore plays a fundamental role in the transmission of genetic information [6,7]. Importantly, it is chemically unstable—it undergoes spontaneous deamination to uracil [8,9]. This process, which occurs with measurable frequency in vivo, leads to the formation of abnormal bases (uracil in DNA) and can result in point mutations if not repaired by appropriate repair enzymes (e.g., DNA glycosylases that remove uracil). In living organisms, cytosine does not occur exclusively in its basic form. Its chemical modifications are common, the most biologically important of which is methylation at C5, leading to the formation of 5-methylcytosine, the so-called fifth base of DNA, which has important epigenetic functions in the regulation of gene expression [10,11]. Methylation of cytosine is widespread in both prokaryotes and eukaryotes, although the extent of this phenomenon varies considerably between species. For example, in mammals 70–80% of cytosines in the context of CpG dinucleotides are methylated, while in yeast or nematodes, DNA methylation is negligible or absent. The discovery of methylcytosine dates back to the early 20th century, as the compound was synthesised already in 1901 and isolated from DNA hydrolysates of *Mycobacterium tuberculosis* in 1925. Subsequent decades of research also revealed other cytosine derivatives in genetic material, including 5-hydroxymethylcytosine (present in the genomes of some bacteriophages and as an intermediate product of DNA demethylation in eukaryotes) or N^4^-methylcytosine found in bacterial DNA [12,13,14].

The purpose of this paper was to review the current knowledge of cytosine and its derivatives, with particular emphasis on analysis using high-performance liquid chromatography (HPLC). The paper discusses the following issues: (1) the occurrence of cytosine and its derivatives in nature; (2) chemical and physicochemical properties of cytosine and its derivatives; (3) main areas of application of these compounds in biology, medicine and industry; and (4) analytical techniques used for their identification and quantification, illustrated by selected examples of research results.

## 2. Occurrence of Cytosine and Its Derivatives in Living Organisms

Cytosine is a component of both DNA and RNA in all organisms. The content of cytosine (or more precisely, GC pairs) in nucleic acids is a species-specific trait and can vary significantly depending on the organism [15,16]. In addition of G:C base pairs in DNA, it has been observed the presence of C:C+ base pairs in C-rich oligonucleotides due to the formation of i-motif structures. The average cytosine content in DNA varies greatly between organisms: from about 10% of all nitrogenous bases (in the genome of the protozoan *Plasmodium falciparum*, which has a very low overall content of GC pairs ~20%) to more than 35% (in the genome of the bacterium *Streptomyces coelicolor*, with GC reaching ~72%). In higher organisms (e.g., humans), cytosine typically accounts for ~20% of all bases (the human genome has an average composition of ~41% GC), while in yeast it accounts for ~19%, and in the intestinal bacterium *E. coli* ~25% (*E. coli* genome~51% GC) [17]. Such large differences result from evolutionarily determined preferences for base pair composition and affect the physical properties of DNA (e.g., the melting point of the helix). In RNA, the cytosine content is usually close to that of guanine, but it can be adjusted depending on the type and function of RNA. Cytosine occurs in RNA, among others, as a component of cytidine in mRNA, rRNA and tRNA [18,19]. Modified nucleosides that are cytosine derivatives, such as 5-methylcytidine or 5-hydroxymethylcytidine, are often found in tRNA, where they play a role in stabilising the structure of tRNA and regulating codon recognition.

Like all pyrimidine and purine bases, cytosine is unlikely to be found in cells in free form (a free heterocyclic compound). It is synthesised and utilised mainly in the form of nucleosides and nucleotides. The biosynthesis of the pyrimidine ring occurs through the stepwise construction of a skeleton from simple metabolites: glutamine, carbon dioxide and aspartate [20,21,22,23,24,25].

## 3. Chemical and Physicochemical Properties of Cytosine

### 3.1. Tautomerism of Cytosine

**Figure 2:**
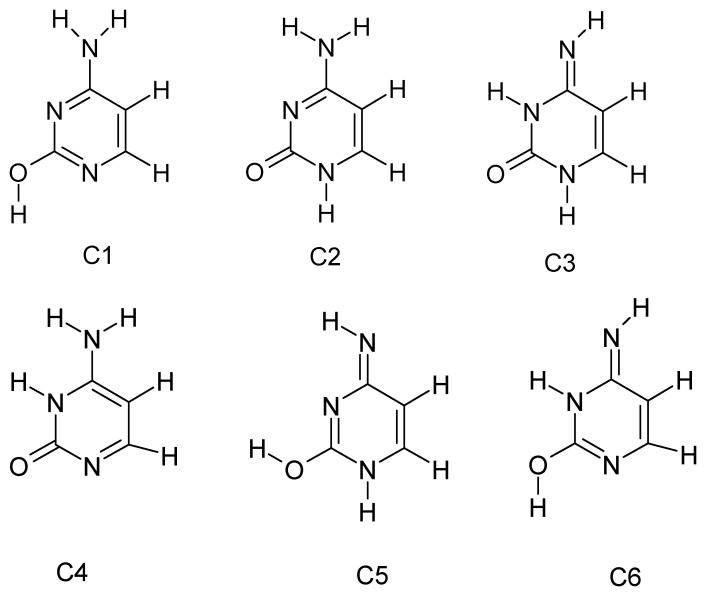
Structural patterns of cytosine tautomers.

As a heterocyclic compound, cytosine occurs in several tautomeric forms [26,27,28,29], resulting from the shift of hydrogen atoms between nitrogen and oxygen atoms in the ring (Figure 2). Six tautomeric forms of cytosine have been identified, three of which (C1, C2, C3) represent the basic structures.

**Figure 3:**
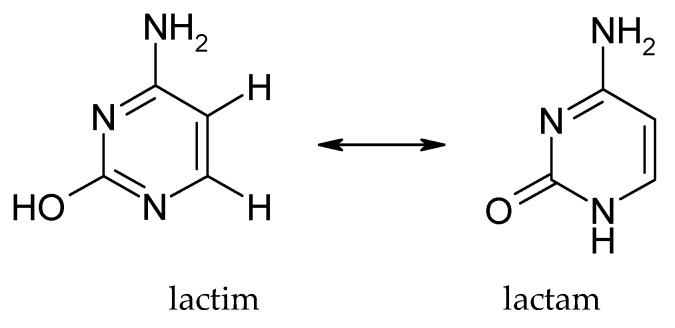
Structural patterns of two forms of cytosine: lactim and lactam [29].

The predominant form under physiological conditions is the 4-amino-2-oxo (lactim) form, in which cytosine occurs as 4-aminopyrimidone (with an –NH_2_ group at C4 and an =O group at C2). Alternative tautomers include the 4-imino-2-oxo form (where the proton from the amino group is shifted to the nitrogen of the ring, forming an =NH group at C4) and the enolic 2-hydroxy-4-amino form (with the proton shifted from N3 to the oxygen atom at position 2). Figure 3 shows a simplified equilibrium diagram of cytosine tautomers.

Due to the favourable bond distribution and aromatic nature of the system, the keto-amino form is the most stable and accounts for the vast majority of cytosine molecules in solution. However, the presence of rarer tautomers is biologically important, as it can lead to abnormal base pairing (e.g., the rare imine tautomer of cytosine can erroneously pair with adenine instead of guanine, which sometimes causes point mutations during DNA replication). The phenomenon of cytosine tautomerism has been studied experimentally, including spectroscopically and crystallographically. As early as the 1970s, a preference for the lactim (hydroxy) form over the lactam (oxo) form was demonstrated. The presence of substituents on the pyrimidine ring can alter the equilibrium of tautomers—for example, fluorine at position 5 (as in 5-fluorocytosine) strongly stabilises the lactim form, while the substitution of more electron-donor groups can favour oxo tautomers. The most abundant tautomeric form of cytosine under physiological conditions is lactim [29].

### 3.2. Acid-Base Properties

Cytosine exhibits amphoteric properties [30,31]—it contains both nitrogen atoms capable of protonation (acting as a Brönsted base) and an –NH_2_ group capable of deprotonation under strongly basic conditions (acting as an acid). There are two degrees of cytosine ionisation in aqueous solution [32]. The first corresponds to the protonation of the N3 atom (a cytosine ion—cation—is formed), with an acidic equilibrium of pKa1≈4.6. This means that at pH below ~4, most of the cytosine molecules are in the cationic (protonated) form, while at neutral pH, the neutral form (non-ionised lactim form of cytosine) predominates. The second degree of dissociation (pKa2≈12.2) is associated with the dissociation of the proton from the 4-NH_2_ group, which occurs under highly alkaline conditions. Above pH~12, only a portion of the molecules take on the anionic (4-amide) form [33]. Under physiological conditions (pH~7), cytosine occurs almost exclusively as a neutral molecule, which is important for base pairing in DNA, as only this form forms correct pairs with guanine. The substitution of electronically active groups affects the acid-base properties of cytosine. For example, 5-fluorocytosine (5-FC) is a much stronger base, because the fluorine substituent reduces pKa1 to about 3.3, which means that 5-FC protonates less readily in aqueous solution (even at slightly acidic pH). At the same time, the electronic effect of fluorine reduces the basicity of the amine group 4-NH_2_, resulting in almost impossible deprotonation of this group (as in uracil, of which 5-FC is an analogue, there is virtually no proton abstraction—the NH group at position 3 of uracil is weakly acidic, pKa~8–9 for 5-FU). The introduction of other substituents (e.g., alkyl groups) generally raises the pKa of the first protonation (making the molecule a stronger base) through an inductive effect; for example, methylation at position 5 slightly increases the basicity of the ring. Knowledge of these parameters is important, among other things, for chromatographic separation conditions, as retention on columns depends on the degree of ionisation of the analytes.

### 3.3. Solubility and Physical Properties

Cytosine, as a pyrimidine base, is a moderately polar compound [34]. One molecule contains three heteroatoms (one oxygen atom and two nitrogen atoms) capable of hydrogen bonds and one new amide group, hence cytosine forms strong hydrogen bonds in the crystal lattice and has a relatively high melting point. In its crystalline form, cytosine forms colourless, shiny plates. It crystallises from water as a monohydrate, losing its hydration water at 100 °C, darkens above 300 °C and decomposes at 320–325 °C. The solubility of cytosine in water at room temperature is limited—1 g dissolves in approx. 130 mL of water (corresponding to ~7–8 mg/mL, or 0.77% *m*/*v*). It can therefore be classified as a compound that is slightly soluble in water. It is practically insoluble in non-polar solvents (e.g., diethyl ether), while it is slightly soluble in lower alcohols (e.g., methanol, ethanol). In aqueous solutions with acidic pH, its solubility increases due to ionisation (the cationic form is better solvated), although at the same time, in strongly acidic environments, slow hydrolysis of the N3-C4 bond can occur (under conditions of concentrated sulphuric acid(VI), cytosine may undergo depurination to uracil) [35,36,37,38]. Cytosine forms salts in reaction with strong acids—for example, with HCl it forms hydrochloride (i.a. crystalline cytosine 5-hydrochloride). In reaction with sodium hypochlorite in the presence of ammonia, cytosine produces a characteristic red colour, which historically was one of the methods used to detect it.

The substitution of a cytosine ring significantly affects solubility [39,40,41]. The general trend is that the introduction of polar groups (e.g., –OH in 5-hydroxymethylcytosine or phosphate in cytidine nucleotides) increases hydrophilicity and solubility in water, while the substitution of non-polar groups (e.g., a methylene group in 5-methylcytosine) decreases solubility. For example, 5-fluorocytosine is more soluble in water than cytosine (about 1.5 g/100 mL, or 15 mg/mL at 25 °C), which facilitates its pharmacological use in the form of oral and intravenous solutions [42,43]. Cytosine nucleosides (cytidine, deoxycytidine) are quite soluble in water (e.g., cytidine ≥ 60 mg/mL in H_2_O) due to the presence of polar sugar groups, and their nucleotides are highly soluble (phosphate salts of nucleotides form compounds that hydrolyse in water) [44]. As a result, cytosine metabolites circulating in the cell are readily soluble in the cytoplasm and easily transported.

### 3.4. Absorption Spectra and Other Properties

Cytosine and its pyrimidine derivatives show characteristic ultraviolet absorption spectra, with maxima associated with electron transitions in the conjugated ring system. For free cytosine in alkaline pH, the maximum absorption occurs at approx. 267 nm (ε≈6.1 × 10^3^ M^−1^cm^−1^) and is stronger in the far UV range at ~197 nm. Cytosine nucleosides and nucleotides have similar spectra (e.g., for CMP, maximum at around 271 nm at neutral pH) [45,46]. These properties are used for practical purposes—measurement of absorbance at 260–270 nm is used for the detection and quantification of cytosine and its derivatives in chromatographic methods (HPLC with UV detection). In aqueous solutions, cytosine is relatively chemically stable under neutral conditions, but it degrades under the influence of UV light (photodegradation via photohydration and deamination reactions) and in extreme pH conditions. The high thermodynamic stability of the pyrimidine ring (aromaticity) of cytosine means that it does not easily open or break down. Only the action of specific enzymes or very strong reactants (e.g., peracetic acid) leads to the destruction of the ring.

#### Properties and Applications of Selected Cytosine Derivatives

Table 1 lists selected cytosine derivatives, both naturally occurring and of pharmacological interest, along with their selected physicochemical properties.

As shown in Table 1, most derivatives of cytosine retain its essential characteristics, i.e., they are polar compounds, usually solids with high melting points (hydrogen bonds in the crystal lattice) and good solubility in water (especially their nucleoside/nucleotide forms), with similar UV spectra. The acid-base parameters remain similar to those of cytosine, with the exception of 5-halogenocytosines (significantly reduced pKa1) and triazine derivatives (5-azacytidine and analogues), where the introduction of an additional nitrogen atom in the ring increases the overall acidity of the compound. In chemical terms, these changes affect stability and reactions: for example, 5-azacytidine is less stable in solution (it is easily hydrolysed, its half-life in water is several hours at room temperature), which is related to the presence of an imino group in the position corresponding to C5. These properties are important for storage and administration of these compounds (5-aza-Cyd must be prepared immediately before use as a drug).

Cytosine is a compound with well-studied chemical properties, providing a reference point for many of its derivatives. Understanding these properties has led to the development of applications for many cytosine derivatives.

## 4. Biological, Medical and Industrial Applications

### 4.1. Biological Role of Cytosine and Its Derivatives

In biology, cytosine plays an indispensable role as an element of the genetic code in DNA and RNA. The sequences of bases in DNA, which also contain cytosine, carry genetic information in all organisms [50,51,52,53,54,55]. The complementary pairing of cytosine with guanine ensures the stability of the double helix and accurate copying of information during replication [56]. Modifications of cytosine in DNA, primarily 5-methylcytosine (5mC), are a key element in the epigenetic regulation of gene activity [57,58,59,60]. In vertebrates, methylation of cytosine within CpG islands (regions in the genome with an increased content of 5′-CpG-3′ dinucleotides relative to the genome average) in the promoters of genes typically silences their expression—this represents a mechanism of cell differentiation and genomic imprinting (one of the mechanisms of epigenetic modification of gene expression in the cell) [61]. The presence of unmethylated CpG in therapeutic oligonucleotides is recognised by the immune system as a ‘foreign DNA signal’, leading to strong activation of the immune response. In contrast, replacing cytosine with its methylated form (5-mC) in CpG blocks recognition by TLR9, thereby preventing unwanted immune activation in therapeutic applications.

Methylation disorders (e.g., hypermethylation of tumour suppressors) lead to diseases, including cancer [62,63], so modulating 5mC levels has become a therapeutic target (e.g., azacitidine as a drug; Table 1). Other natural derivatives, such as 5-hydroxymethylcytosine (5hmC), discovered in mammalian neurons, among others, probably act as mediators of active DNA demethylation and may themselves act as epigenetic signals [64,65,66,67,68]. In bacterial DNA, N4-methylcytosine can be found, which is formed by the action of restriction methylases and protects the bacteria’s own DNA from degradation by the restriction–modification system (foreign DNA lacking this methylation is recognised and cut) [69]. In RNA, on the other hand, cytosine is included in all major types of RNA (mRNA, tRNA, rRNA, snRNA). Its modifications in RNA (e.g., 5-methylcytosine in tRNA and rRNA) stabilise secondary and tertiary structures and affect translation efficiency [70,71].

In terms of metabolism, cytidine triphosphate (CTP) is not only a precursor of RNA, but also acts as a coenzyme—for example, CTP is a donor of phosphate residues in nucleoside phosphorylation reactions (CTP can transfer phosphate to ADP in the presence of specific kinases, converting it to ATP) [72]. In addition, cytidine nucleotides are involved in the biosynthesis of cell membrane phospholipids, with CTP reacting with choline to form CDP-choline (cytidine diphosphocholine, known as citicoline), which is a key intermediate in the synthesis of phosphatidylcholine [73,74,75]. Citicoline is sometimes used as a dietary supplement or agent in neurorehabilitation after stroke, providing the body with cytidine and choline to support the regeneration of neuronal membranes [76].

### 4.2. Medical Applications as Cytosine Drugs

**Figure 4:**
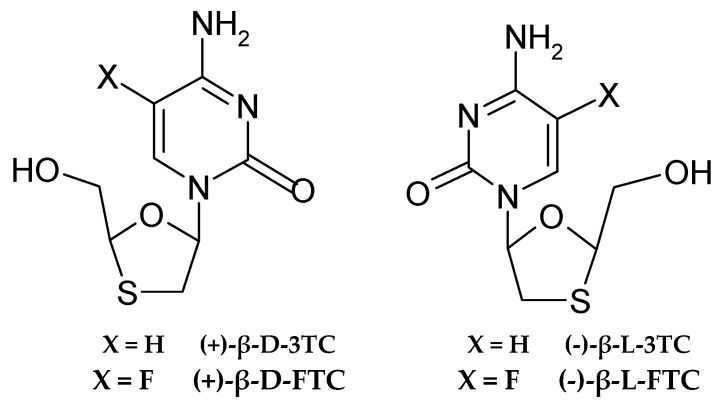
Structural of FTC izomers.

**Figure 5:**
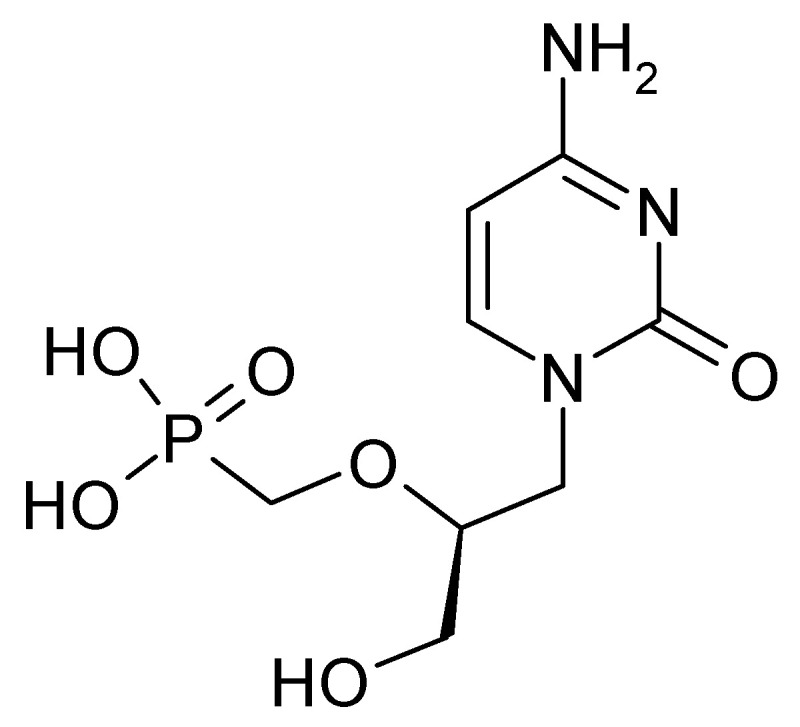
Structural of *cidofovir*.

Minor structural changes in the cytosine molecule result in significant changes in physicochemical properties, ranging from tautomeric ability, solubility and chemical stability to affinity for metabolizing enzymes. These differences translate into varied biological activities of cytosine derivatives. Pure cytosine as a free base has no direct pharmacological activity, but it is a key biological element, as its methylation (5mC) in DNA is a fundamental epigenetic marker regulating gene activity [77,78]. Much more interesting is the biological activity of cytosine derivatives, many of which have found application as anticancer drugs, antiviral agents or antibiotics. Cytosine has been reflected in the development of many drugs that are analogues of cytosine or its nucleosides [79,80]. Due to the involvement of cytosine in critical processes (DNA replication, RNA transcription), its analogues most often act as antimetabolites, disrupting these processes in pathogens or cancer cells [81,82]. The most important examples include:

- Anticancer drugs (antimetabolites): Cytarabine (Ara-C, cytosar) is 1-β-D-arabinofuranosylcytosine, a cytidine analogue containing arabinose instead of ribose [83,84]. It was introduced for therapeutic use in the 1960s (approved by the FDA in 1969), it became the primary drug for treating acute leukaemia (especially acute myeloid leukaemia). When phosphorylated to ara-CTP, cytarabine is incorporated into DNA instead of deoxycytidine and causes inhibition of DNA chain elongation and polymerase function, leading to the death of rapidly dividing cancer cells [85]. Gemcitabine (difluorodeoxycytidine, dFdC) is another cytostatic, a deoxycytidine analogue, in which the two hydrogen atoms at C2′ of the sugar have been replaced by fluorine [86,87,88]. Introduced in oncology in the 1990s (approved by the FDA in 1996 as a first-line drug for pancreatic cancer), gemcitabine has also been used in the treatment of non-small cell lung cancer, bladder cancer, breast cancer and other cancers [89,90,91]. Its mechanism involves a dual action: in the form of triphosphate, it is incorporated into DNA, causing termination of synthesis, while gemcitabine diphosphate inhibits ribonucleotide reductase, reducing the pool of deoxyribonucleotides in the cell. Other important drugs are 5-azacytosine analogues: 5-Azacytidine (AZA, Vidaza^®^) and 5-aza-2′-deoxycytidine (decitabine, Dacogen^®^). These are nucleosides in which the C5 carbon atom of pyrimidine is replaced by nitrogen (the ring becomes a triazine). They incorporate into DNA (decitabine and azacitidine) or RNA (only azacitidine) and, due to the presence of nitrogen at position 5, are not subject to methylation by DNA methyltransferases [92,93]. Furthermore, they form covalent adducts with these enzymes, leading to their degradation. As a result, these drugs cause global DNA hypomethylation and re-activation of silenced suppressor genes in cancer cells. They were approved for the treatment of myelodysplastic syndromes (MDS) and certain types of leukaemia around 2004. Although they act atypically (not so much cytotoxically as epigenetically), they improve survival of MDS patients and are being intensively studied in combination with other therapies. Other cytidine analogues are also used in chemotherapy, such as *azacitidine* (liposomal DepoCyt^®^ for the treatment of CNS lymphomas) or *gemcitabine* (discussed above) [94,95]. New derivatives are also being developed, such as olutasydenib (FT-2102), a decitabine analogue with a modified structure, or RX-3117 (fluorocyclopentenylcytosine), a cytidine analogue active in some resistant cancers [96].
- Antiviral drugs: The structure of cytosine nucleosides provided the basis for the development of effective drugs against viruses, especially retroviruses and hepatitis B virus. *Lamivudine* (3TC) is a 2′,3′-dideoxy-3′-thiazolidinedione analogue of deoxycytidine, in which the ribose ring was replaced by a ring with a sulphur atom (thiazolidine), and the absence of 3′-OH groups prevents DNA chain elongation [97]. Lamivudine was approved for the treatment of HIV-1 infections in 1995 and HBV in 1998, and has become a widely used component of antiretroviral therapy (it belongs to the NRTI class of nucleoside reverse transcriptase inhibitors) [98,99]. It acts as a terminator of viral DNA synthesis after incorporation by reverse transcriptase, while competitively inhibiting the enzyme itself. *Emtricitabine* (FTC), a fluorinated analogue of lamivudine (Figure 4), also used in the treatment of HIV—*human immunodeficiency virus* (often in composite formulations), has a similar effect.
- Another example is *cidofovir*—although it is a nucleotide (phosphate) analogue of cytosine (Figure 5) rather than a nucleoside, it exhibits activity against many DNA viruses (CMV—*Cytomegalovirus*, adenoviruses, polio) by inhibiting viral DNA polymerase. It is used in CMV retinal infections [100,101].
- In the context of new threats, the pro-drug *molnupiravir* (EIDD-2801) was emergency approved in 2021 for use against SARS-CoV-2 [102,103]. It is a modified ribonucleoside derivative of cytosine (N^4^-hydroxycytidine in the form of a pro-drug) which, when activated to triphosphate, incorporates into the virus RNA, causing lethal mutations in its genome. This is an example of the use of a cytosine derivative to induce viral replication errors. In the treatment of DNA viruses (such as HSV—*herpes simplex virus*), cytosine derivatives play a lesser role, and guanine analogues (acyclovir) are better known [104,105]. Nevertheless, work on cytidine analogues with activity against RNA and DNA viruses continues.
- Antifungal drugs: The only commonly used antifungal antimetabolite is the aforementioned *5-fluorocytosine* (flucytosine, 5-FC) [106,107]. Introduced for the treatment of fungal infections in the 1970s, it is still used today (mainly in combination with amphotericin B) to treat cryptococcal meningitis and other severe fungemia [108,109]. 5-FC has no direct toxic effect on mammalian cells, as they are unable to metabolise it. In fungal cells, however, cytosine deaminase (enzyme) converts 5-FC to 5-fluorouracil (5-FU). 5-FU, in turn, is incorporated into RNA instead of uracil and inhibits thymidylate synthase (after conversion to 5-F-dUMP), which disrupts DNA synthesis. This results in a fungistatic (in higher doses fungicidal) effect of 5-FC. This drug is a valuable addition to therapy, although due to the rapid development of resistance (deaminase or pyrimidine permease mutations), it is mainly used in polytherapy. Flucytosine is an example of how a minor change in the molecule (a fluorine atom instead of hydrogen in the cytosine ring) adds a completely new pharmacological use to a compound.
- Other pharmacological uses: In addition to the examples discussed above, it is worth mentioning that cytidine and its phosphates are present in dietary supplements and products designed to enhance brain function [110]. The aforementioned citicoline (CDP-choline, which is cytidine diphosphate coupled with choline) is available as a preparation that improves cognitive function and accelerate neuronal regeneration after strokes. Its mechanism of action involves increasing the availability of cytidine (or rather uridine, which is produced from cytidine) and choline, precursors of the synthesis of important brain phospholipids. Another example is *carmofur*, a 5-FU derivative coupled with a carbamate residue, which also exhibits anticancer activity (although it is not a pure analogue of cytosine, but rather its metabolite) [111]. An interesting fact is the use of cytosine analogues in biotechnology, e.g., for DNA labelling. The 5-bromocytosine can be incorporated in place of cytosine and then used for specific DNA cleavage with UV light (photochemically). In medical diagnostics, the 5mC methylation profile of a patient’s genome (the so-called methylome map) is analysed using chemical conversion of cytosine to uracil by the so-called bisulfide reaction. The reagent is sodium metabisulfite, which selectively deaminates cytosine to uracil, while 5-methylcytosine remains unaffected. This technique allows the methylation pattern to be read after DNA sequencing and is routinely used in epigenetics.

In summary, chemical modifications of cytosine lead to compounds with diverse biological activities. From nucleoside analogues that disrupt viral and cancer cell replication, through pro-drugs activated in microorganisms, to natural antibiotics, cytosine derivatives represent an important class of bioactive compounds in medicine and biology.

### 4.3. Industrial and Technological Applications

Apart from medicine and molecular biology, cytosine derivatives do not have very broad independent industrial applications. However, the production of nucleosides (including cytidine) and nucleotides has become important in the biotechnology and pharmaceutical industries. Cytidine is produced by fermentation or extraction (from yeast) as a raw material for the synthesis of the aforementioned citicoline or as a supplement. CTP and CDP-choline are used in nucleotide-enriched food preparations (e.g., some infant formulae contain nucleotide additives, including CMP, although adenosine and guanosine nucleotides are more commonly added as umami flavour enhancers) [74,75]. Cytosine derivatives interact with guanosine, most often maintaining the classic C–G hydrogen bond arrangement, but the nature of this interaction and its stability depend on the type of cytosine modification.

In biochemical laboratories, it is standard practice to use isotopically labelled derivatives of cytosine, such as tritiated cytidine (^3^H), to study metabolic pathways, DNA/RNA synthesis, etc. Synthetic derivatives are also produced on an industrial scale for scientific purposes, e.g., 5-ethynyl-2′-deoxycytidine (EdC), a nucleoside used for labelling newly synthesised DNA (so-called click chemistry in the detection of proliferating cells) [112,113]. In summary, the extra-cellular applications of cytosine are mainly in the specialised fields of biotechnology, diagnostics and pharmaceutical production, while its biological role is universal and crucial for life.

## 5. Analytics of Cytosine and Its Derivatives

The analysis of cytosine and its derivatives plays an important role both in basic research (e.g., determining the composition of bases in DNA, studying nucleotide metabolism) and in therapy monitoring (determining the concentration of drugs—cytosine analogues in the patient’s blood). Classical identification methods include colour reactions (e.g., Sakaguchi test modified for pyrimidines) and blotting or thin-layer chromatography (TLC) for separating mixtures of nitrogenous bases. Today, high-performance liquid chromatography (HPLC) and capillary electrophoresis techniques are standard, often combined with UV spectrophotometric detection or mass spectrometry (MS) methods. The HPLC technique is the most widespread in the analysis of nucleosides and their analogues.

### 5.1. High-Performance Liquid Chromatography (HPLC)

The analysis of cytosine and its derivatives requires sensitive and selective methods capable of separating structurally very similar compounds. High-performance liquid chromatography is a widely used analytical technique for the determination of both free nitrogenous bases, nucleosides and their analogues in a variety of matrices (pure solutions, DNA hydrolysates, biological samples or pharmaceutical preparations) [114,115,116,117]. To achieve proper separation of structurally similar compounds, attention must be paid to key aspects of the HPLC methods used and the conditions of analysis:

- Columns and stationary phases: The most commonly used are strongly hydrophobic C18-type (reversed-phase) columns with high inertness, allowing the separation of polar nucleosides in the water-organic solvent system [118,119]. However, separation of very similar cytosine analogues (e.g., 5 different epigenetic derivatives: C, 5mC, 5hmC, 5fC, 5caC) can sometimes be challenging. In such cases, modification of the stationary phase can improve selectivity. For example, using a phenyl-hexyl column instead of C18 increased the differences in cytosine vs. 5-hydroxymethylcytosine retention due to π-π interactions. This allowed for the separation of all five cytosine analogues associated with DNA demethylation, which was not achieved on the classical C18 column [120]. In the analysis of highly polar derivatives, such as phosphate nucleotides, an ion-pairing reagent (e.g., tetrabutylammonium iodide) is often added to the mobile phase, or ion-exchange or HILIC (hydrophilic interaction chromatography) columns are used [121]. These approaches increase the retention of ionic or highly polar compounds on the column.
- Mobile phases: Buffered aqueous phases (e.g., ammonium or potassium phosphate buffer solutions with pH in the range of 4–7) combined with an organic solvent (methanol or acetonitrile) are typically used for the separation of cytosolic bases and nucleosides. The pH of the mobile phase is critical because it affects the degree of ionisation of analytes and interactions with the stationary phase [120,122]. Studies have shown that changes in pH induce the largest changes in the retention of cytosine and cytidine. A slightly acidic pH is usually maintained (approximately 4–6) to ensure that cytosine (pKa~4.5) is present in an ionised or partially ionised form, which reduces its retention and improves the shape of peaks. Elution gradients are often used in the analysis of mixtures of derivatives with significantly different polarities: for example, starting with a high proportion of water (or buffer) for the retention of polar nucleosides, and then gradually increasing the proportion of organic solvent to elute more non-polar analogues. This method was used to separate a mixture of cytosine and its modified derivatives, starting with 1% methanol in water with a buffer and reaching 30% methanol in several minutes, achieving excellent separation of all five analytes in <12 min [120].
- Detectors and sensitivity: Cytosine derivatives have an aromatic system, so they strongly absorb UV light in the 260–280 nm range. The most common detection technique is therefore UV detection (often using a DAD, i.e., diode array detector), set, e.g., at 270–280 nm for cytosine nucleosides. UV detection usually allows for the detection of nanomolar amounts of analyte [123,124,125]. For example, at a wavelength of 271 nm, the detection limit for cytidine in pharmaceutical analysis was about 0.15 ng and the limit of quantification was ~0.5 ng [126]. The sensitivity of HPLC–UV can be improved by increasing the injection volume (up to several dozen μL, if tolerated by the column) and by using sample extraction (analyte concentration) from the matrix. For applications requiring ultra-high sensitivity or selectivity (e.g., determination of trace modifications of bases in cellular DNA), mass spectrometry coupled with HPLC (LC-MS/MS) is used. The HPLC-ESI-MS/MS technique with selected reaction monitoring (MRM) enabled the simultaneous quantification of 5-methyl- and 5-hydroxy-2′-deoxycytidine alongside unaltered deoxycytidine with a detection limit of 0.5 femtomoles (corresponding to the analysis of 50 ng of hydrolysed genomic DNA, enabling the detection of 0.1% 5hmC content) [127]. Such high sensitivity allows for precise profiling of global DNA methylation in biological samples.

In summary, cytosine, cytidine nucleosides and their derivatives are polar compounds that are difficult to separate on classical inverted columns (C18) without modifying the mobile phase. Under neutral conditions, these molecules are poorly separated on the non-polar phase (due to hydrophilicity), while under acidic conditions they become cations, which further reduces retention due to repulsion from the silanol groups remaining on the silica. Very good HPLC analysis results can be obtained by: (1) adding ion-pairing reactants to the mobile phase (e.g., alkyl sulfonates), which form ion pairs with cationic molecules that are more easily retained on the C18 column; (2) using an ion-exchange phase, such as a cation-exchange column for retaining pyrimidine cations; (3) using specialised columns for hydrophilic polar interaction chromatography or mixed columns with polar-ion groups.

A modern solution is the so-called BIST (Bridge Ion Separation Technology), which combines ionic and hydrophobic retention. An example is the method developed by SIELC Technologies for separating a mixture of cytosine, deoxycytidine and cytidine on a BIST B+ column. Under the conditions of this method (mobile phase: acetonitrile/water mixture with 0.2% H_2_SO_4_, column with quaternary amine groups on the surface), separation of three compounds was achieved in ~5 min, with retention times of approximately 2.8; 3.2 and 5.1 min, respectively. Sulphuric acid provides protonation of the analytes and the formation of ‘ion bridges’ with the stationary phase, resulting in significant differences in retention despite similar structures [128].

In a classical RP-HPLC system (C18 phase, UV detector), separation of pyrimidine nucleosides is also possible, but requires precise control of the pH and composition of the mobile phase. A study by Romanova and Novotny (1996) [129] showed that the most important factor affecting cytosine and cytidine retention is the pH of the eluent. A change in pH from 5 to 7 caused a drastic increase in retention time due to the transition of cytosine from the cationic to the neutral form [129]. In contrast, increasing the content of methanol in the mobile phase shortened retention times (as expected for revered-phase chromatography), while changes in temperature and flow had a lesser effect. With such optimisations, it is possible to separate mixtures of nucleosides by RP–HPLC in several minutes. For example, cytosine, uracil, cytidine, deoxycytidine and other metabolites can be separated in a single run of gradient HPLC with phosphate buffer and methanol [130]. UV detection at 254–270 nm allows for picomole sensitivity for individual compounds. In addition, coupling HPLC with a mass detector (LC–MS) allows unambiguous identification of analysed derivatives on the basis of their mass spectra [131,132]. In the case of cytostatic analogues (e.g., cytarabine, gemcitabine), LC–MS/MS is routinely used to monitor pharmacokinetics, as these compounds are detected in plasma at nanomole concentrations due to specific fragmentations in the spectrometer. For example, gemcitabine (mass 264u) contains a characteristic fragment (mass 112, after sugar loss), which allows for sensitive quantitative determination in patients receiving therapy [133].

**Figure 6:**
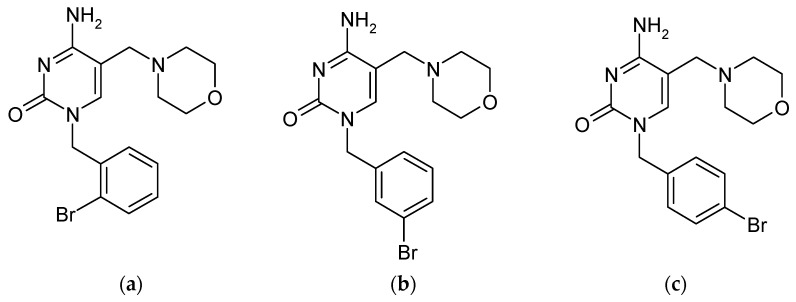
Structures of: (**a**) 4-amino-1-(2-bromobenzyl)-5-(morpholinomethyl)pyrimidin-2(1*H*)-one, (**b**) 4-amino-1-(3-bromobenzyl)-5-(morpholinomethyl)pyrimidin-2(1*H*)-one, and (**c**) 4-amino-1-(4-bromobenzyl)-5-(morpholinomethyl)pyrimidin-2(1*H*)-one.

The authors of this article also studied the separation of isomers of cytosine derivatives. The study involved the separation of cytokine derivative isomers in the form of three isomeric bromine derivatives [134], which are N1-substituted benzylcytosines (differing in the position of the bromine in the benzyl ring; Figure 6).

**Figure 7:**
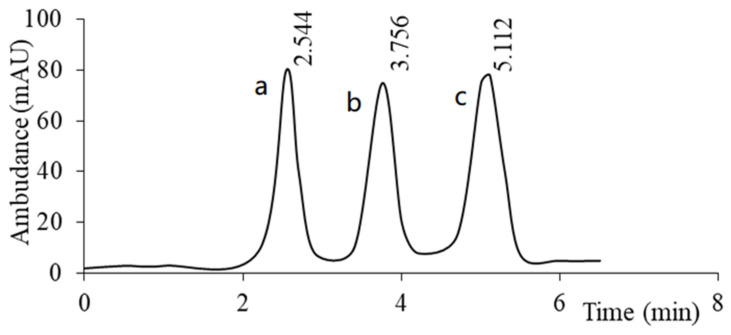
An example of a chromatogram of the separation of (**a**) 4-amino-1-(2-bromobenzyl)-5-(morpholinomethyl)pyrimidin-2(1*H*)-one, (**b**) 4-amino-1-(3-bromobenzyl)-5-(morpholinomethyl)pyrimidin-2(1*H*)-one, and (**c**) 4-amino-1-(4-bromobenzyl)-5-(morpholinomethyl)pyrimidin-2(1*H*)-one.

Attempts at separation on standard C18 and C8 phases were unsuccessful as the isomers eluted together regardless of the composition of the mobile phase. Only the use of a column with a chemically attached naphthylpropyl ligand (aromatic phase) enabled the effective separation of these isomers in less than 7 min [135]. The aromatic phase promotes isomer differentiation through specific π-π interactions and selective geometric matching [136,137]. The authors achieved high separation selectivity (coefficients α = 1.90 and 1.53 between consecutive isomers; Figure 7). The aforementioned chemically bonded naphthylpropyl stationary phase was successfully used to separate a wide variety of derivatives with similar structures containing an aromatic ring [138,139,140].

In summary, the above examples show that even structurally very similar derivatives can be separated chromatographically with proper optimisation, which is important, for example, in drug quality control, where individual isomers or synthesis by-products must be detected and determined. The HPLC technique is an indispensable tool in the analysis of cytosine and its derivatives. It allows the separation of even structurally very similar analogues by selecting the appropriate column and elution conditions, and in combination with sensitive detectors (UV-DAD, MS/MS) enables the detection of trace amounts of these compounds in complex matrices. HPLC techniques are used in quality control of drug substances (e.g., purity and stability of cytosine analogues in pharmaceutical preparations), in monitoring the pharmacokinetics of anticancer and antiviral drugs (determination of plasma concentrations), as well as in basic research [141]. With the continuous development of chromatography, such as the use of ultra-high performance liquid chromatography (UHPLC) and improvements in MS detection, the scope and precision of analyses of cytosine and its derivatives continues to expand, supporting advances in chemistry, biology and medicine.

### 5.2. Chromatography and Electrophoresis for DNA/RNA Studies

In laboratory analysis of DNA base composition, separation of the products of total nucleic acid hydrolysis is commonly used. DNA hydrolysed by acid (or enzymatically by nucleases to a mixture of nucleosides) can be analysed by reversed-phase HPLC. Individual deoxyribonucleosides (dA, dG, dC, dT) elute in a specific order and are detected spectrophotometrically. This allows, for example, the determination of the overall level of methylation (by comparing the ratio of dC to 5mdC). In research practice, tandem MS is used to determine modified bases (such as 5hmC, 5fC, 5caC) due to their very low amounts in DNA. It is worth noting that commercial HPLC columns specifically designed for nucleotide separation are available. They often use ion exchange mechanisms, e.g., with amine groups. An alternative method is high-performance capillary electrophoresis (HPCE)—a high-resolution technique where nucleosides are separated based on differences in charge and size in an electric field [142]. It has been successfully used to analyse mixtures of nitrogenous bases and nucleosides, although it requires UV detection in the far ultraviolet (which is sometimes less sensitive).

### 5.3. Other Analytical Methods

In addition to HPLC, spectroscopic methods are also used in the analysis of cytosine and its derivatives. UV-VIS spectroscopy is used for rapid determination of the total content (e.g., measurement of absorbance at 260 nm gives the total content of a mixture of nucleic bases, used for determining DNA/RNA concentration) [143,144]. Fluorescence spectroscopy has limited application because cytosine and nucleosides are not naturally fluorescent. However, it is possible to determine them indirectly after labelling with dyes, e.g., using Hantzsch’s reagent, which forms a fluorescent derivative of ureides, a technique used in early methods of RNA determination. A highly sensitive method is GC-MS after prior derivatisation of bases into volatile derivatives, e.g., cytosine forms trimethylsilyl derivatives, which can be separated by gas chromatography and detected by mass spectrometry. This technique has been used to study prebiotic experiments (searching for cytosine in abiotic mixtures). Finally, the aforementioned enzymatic methods are popular in molecular biology. For example, cytosine deaminase can be used for the differential determination of 5mC vs. C (5mC is not a substrate for the enzyme). Overall, however, high-performance liquid chromatography with various modifications remains the ‘gold standard’ for the analysis of cytosine and derivatives, from simple base separation to complex mixtures of pharmaceutical analogues. Its advantage is that it combines UV detection (universal for aromatic compounds) and MS (for selectivity) to provide reliable identification and quantitative precision necessary in science and diagnostics [145].

## 6. Conclusions

Cytosine is not only an elementary component of nucleic acids, but also a chemical platform for many compounds of great biological and medical importance. Its chemical properties, i.e., an aromatic pyrimidine ring capable of tautomerism, moderate basicity and modifiability, determine both the stability of genetic information and the possibility of subtle epigenetic regulation through methylation. Cytosine derivatives play key roles in gene regulation (5-mC, 5-hmC), and their synthetically produced analogues have become powerful tools for treating diseases (antimetabolites—cytarabine, gemcitabine, azacitidine; antiretroviral drugs—lamivudine, etc.). Understanding the metabolism of cytosine (biosynthesis from UTP, degradation to β-alanine) has provided a foundation of biochemical knowledge, while the development of analytical methods has made it possible to track its fate in the cell and the therapeutic effects of its analogues. Modern analytics, especially HPLC techniques, enable the effective separation and determination of cytosine and its derivatives, even in complex biological matrices, which is essential, for example, in epigenomics (5-mC determination) or drug pharmacokinetics. Various research works show that methods for separating even very similar structures of cytosine derivatives are constantly being improved, which is important for the development of new drugs and quality control. In summary, cytosine is a crucial nexus between chemistry and biology—from the very beginnings of life (the problem of cytosine abiogenesis), through genetic and epigenetic mechanisms, to the design of life-saving drugs. Its numerous derivatives, both natural and synthetic, expand the range of functions and applications of this seemingly “ordinary” nucleotide base. Thanks to intensive research on cytosine, we now have both a profound understanding of biological processes and specific tools for modelling them in medicine and biotechnology.

## Figures and Tables

**Table 1:** Selected cytosine derivatives—properties and applications [29,47,48,49].

Compound (Abbreviation)	Structure (Modification)	pK_a_ (≈)	Melting Point	Solubility in H_2_O (25 °C)	Applications/Remarks
Cytosine (C)	4-aminopyrimidin-2(1*H*)-one	~4.6; 12.2	320–325 °C ^#^	~7–8 mg/mL	DNA/RNA principle; undergoes methylation to 5-mC
5-Methylcytosine (5-mC)	cytosine with –CH_3_ in position 5	4.6; 12.4 (estimate)	270 °C ^#^	poorly soluble < 5 mg/mL (estimate)	Epigenetic modification of DNA (gene regulation)
5-Fluorocytosine (5-FC)	cytosine with –F in position 5	~3.3; ~11	295–297 °C ^#^	15 mg/mL	Antifungal drug (5-FU prodrug)
5-Azacytosine (in 5-aza-Cyd)	pyrimidine with N instead of C5	~1.1; 9.2 (for 5-azaC)	>300 °C	highly soluble (in nucleoside form)	Component of azacitidine and decitabine drugs (DNA hypomethylation)
Cytidine (Cyd)	nucleoside: cytosine + ribose β	4.2; ~12.5 (principle)	230.5 °C ^#^	≥60 mg/mL	RNA component; supplement (citicoline)
Deoxycytidine (dCyd)	nucleoside: cytosine + 2′-deoxyribose	4.3; ~12 (principle)	207–210 °C ^#^	highly soluble (≥50 mg/mL)	DNA component
5-Azacytidine (5-aza-Cyd)	cytidine analogue with C5 → N (triazine)	-	~229 °C ^#^	≥50 mg/mL (water, unstable)	Anti-cancer drug (Vidaza^®^; MDS, leukaemia)
Decitabine (5-aza-dCyd)	deoxycytidine analogue (C5 → N)	-	209 °C ^#^	good solubility in water	Anti-cancer drug (Dacogen^®^; MDS)
Cytarabine (Ara-C)	1-β-D- arabinofuranosylcytosine	4.2; 12 (principle)	212–213 °C ^#^	~10 mg/mL (water)	Cytostatic drug (Ara-C; leukaemia)
Gemcitabine (dFdC)	2′,2′-difluorodeoxycytidine	3.6; 12 (principle)	~168 °C ^#^	~15 mg/mL (water)	Cytostatic drug (Gemzar^®^; pancreatic cancer, others)
Lamivudine (3TC)	2′,3′-dideoxy-3′-thiacytidine (S in the ring)	~4; 12 (principle)	160–162 °C	≥20 mg/mL (water)	Antiretroviral drug (NRTI; HIV, HBV)

^#^—decomposition.

## Data Availability

No new data were created or analyzed in this study. Data sharing is not applicable to this article.
